# Comprehensive subcellular topologies of polypeptides in *Streptomyces*

**DOI:** 10.1186/s12934-018-0892-0

**Published:** 2018-03-15

**Authors:** Konstantinos C. Tsolis, Evridiki-Pandora Tsare, Georgia Orfanoudaki, Tobias Busche, Katerina Kanaki, Reshmi Ramakrishnan, Frederic Rousseau, Joost Schymkowitz, Christian Rückert, Jörn Kalinowski, Jozef Anné, Spyridoula Karamanou, Maria I. Klapa, Anastassios Economou

**Affiliations:** 1 Laboratory of Molecular Bacteriology, Dpt. of Microbiology and Immunology, Rega Institute, KU Leuven, Herestraat 49, 3000 Leuven, Belgium; 2 Metabolic Engineering & Systems Biology Laboratory, Institute of Chemical Engineering Sciences, Foundation for Research and Technology-Hellas (FORTH/ICE-HT), Patras, Greece; 3 Department of General Biology, School of Medicine, University of Patras, Patras, Greece; 4 Institute of Molecular Biology and Biotechnology-FoRTH, P.O. Box 1385, Iraklio, Crete Greece; 5 Center for Biotechnology (CeBiTec), Universität Bielefeld, 33594 Bielefeld, Germany; 6 VIB-KU Leuven Center for Brain & Disease Research and VIB Switch Laboratory, Department for Cellular and Molecular Medicine, KU Leuven, Herestraat 49, 3000 Leuven, Belgium

**Keywords:** *S. lividans* TK24, Proteome annotation, Protein subcellular topology, Protein subcellular localization, Database, Secretome, Membranome, Signal peptide, Sortase, Peptidoglycan, Sec system, TAT system

## Abstract

**Background:**

Members of the genus *Streptomyces* are Gram-positive bacteria that are used as important cell factories to produce secondary metabolites and secrete heterologous proteins. They possess some of the largest bacterial genomes and thus proteomes. Understanding their complex proteomes and metabolic regulation will improve any genetic engineering approach.

**Results:**

Here, we performed a comprehensive annotation of the subcellular localization of the proteome of *Streptomyces lividans* TK24 and developed the Subcellular Topology of Polypeptides in *Streptomyces* database (SToPSdb) to make this information widely accessible. We first introduced a uniform, improved nomenclature that re-annotated the names of ~ 4000 proteins based on functional and structural information. Then protein localization was assigned de novo using prediction tools and edited by manual curation for 7494 proteins, including information for 183 proteins that resulted from a recent genome re-annotation and are not available in current databases. The *S. lividans* proteome was also linked with those of other model bacterial strains including *Streptomyces coelicolor* A3(2) and *Escherichia coli* K-12, based on protein homology, and can be accessed through an open web interface. Finally, experimental data derived from proteomics experiments have been incorporated and provide validation for protein existence or topology for 579 proteins. Proteomics also reveals proteins released from vesicles that bleb off the membrane. All export systems known in *S. lividans* are also presented and exported proteins assigned export routes, where known.

**Conclusions:**

SToPSdb provides an updated and comprehensive protein localization annotation resource for *S. lividans* and other streptomycetes. It forms the basis for future linking to databases containing experimental data of proteomics, genomics and metabolomics studies for this organism.

**Electronic supplementary material:**

The online version of this article (10.1186/s12934-018-0892-0) contains supplementary material, which is available to authorized users.

## Background

Members of the genus *Streptomyces* are soil-dwelling Gram-positive bacteria, belonging to the phylum *Actinobacteria* with significant industrial and academic interest [1, 2]. Today, most of the secondary metabolites used in industry and medicine are produced in *Streptomyces* spp. [3, 4]. In addition, *Streptomyces* strains have been used as cell factories for heterologous protein expression, including interleukin-6 (IL-6) [5], human mature form of interferon alpha 2 (hIFN-alpha 2) [6], mouse tumor necrosis factor alpha (mTNF-alpha) [7], Xeg glucoside hydrolase from *Jonesia* sp. [8] and a thermophilic cellulase from Rhodothermus [9]. There are two widely used model strains in this family: *Streptomyces coelicolor* A3(2), used for the production of antibiotics and secondary metabolites [10], and its close relative *Streptomyces lividans*, which is utilized for heterologous protein production [11]. In our study, we focus primarily on *S. lividans* strain TK24 which has been used for more than 25 years for protein expression and secretion [12–14].

*Streptomyces lividans* has several advantages that motivate its use as an expression strain. As a Gram-positive bacterium without a mycolic acid layer, the single cell membrane allows protein secretion of folded proteins directly to the culture medium. This avoids accumulation in the periplasm and formation of inclusion bodies seen in *E. coli* cells. Compared to many other *Streptomyces* strains, *S. lividans* TK24 is attractive as a host for genetic manipulation, because it is insensitive to methylated DNA, has a relatively low endogenous protease activity and a collection of vectors and promoters are already available [13]. *Streptomyces* species export a large amount of their native proteins, including several hydrolases [15], using both the Sec secretion pathway for secretion of nascent, unfolded polypeptides [16] and the TAT pathway for secretion of folded substrates [17] and to a lesser extent the Type VII system [18]. These proteins collectively comprise the “secretome”. Another component of the exportome, i.e. the total number of non-cytoplasmic polypeptides, is the membranome that comprises polypeptides with transmembrane regions (TM) that are embedded in the plasma membrane of the cell and many of them are involved in transport and sensing. In addition to native proteins, several recombinant proteins have been produced as secretory proteins [13]. However, several parameters, including growth conditions, affect protein production and perhaps secretion, resulting in variable yields of protein recovery in the spent growth medium [14, 19]. It becomes apparent that better understanding of how protein secretion is regulated in *S. lividans* TK24 is essential for the broader use of this strain in protein secretion biotechnology.

Comprehensive protein topological information is not available in the currently available databases specialized for *Streptomyces* such as StreptomeDB (http://www.pharmaceutical-bioinformatics.org/streptomedb/) that focuses on natural products produced by different *Streptomyces* species [20] and StrepDB (http://streptomyces.org.uk/) that includes genome annotation information for multiple *Streptomyces* species. The main access to the latter database is through custom made scripts and is therefore limited for the wider user community. Topology annotation in Uniprot was the result of automated, non-curated annotation and assigned topologies to only 27% of the proteome. In addition, LocateP provides and automated subcellular topology prediction tool for Gram-positive bacteria with an accuracy > 90%, which due to automatic scoring algorithms includes misclassified proteins comparing with the individual specialized prediction tools [21].

To provide a generally accessible protein subcellular topology tool, we developed the Subcellular Topology of Polypeptides in *Streptomyces* (SToPSdb) database, containing information of proteome subcellular topology and protein annotation for TK24. Protein localization was assigned using a combination of bioinformatic tools for protein localization and structural motif prediction, similarity-based alignments (BLAST) and available literature. We used five localization prediction tools and conflicting predictions were edited manually. Protein names were re-annotated providing a more detailed description of protein function. This re-annotation is based on the protein amino acid similarity to known proteins of other *Streptomyces* species as well as the presence of characteristic structural domains identified through search in the corresponding databases. The content of the SToPSdb database is regularly updated based on experimental and newly published data. The structure of SToPSdb was based on the protein localization database for *E. coli*, STEPdb that we developed previously [22] and on all the formalisms of Uniprot so that, in effect, information entered in SToPSdb can be easily transferred to Uniprot in future updates. We also expect to link SToPSdb to databases that store experimental data for the multi-omics characterization of *S. lividans* and its reconstructed metabolic and protein–protein interaction models, towards a comprehensive resource for this bacterium. In a first effort in this direction SToPSdb incorporates topology-related proteomics data currently available.

## Methods

### Data collection and assignment of subcellular topologies

The recently re-annotated genome sequence of *S. lividans* strain TK24 was used as reference (Busche et al. in preparation). Identified ORFs were translated into proteins and protein localization was assigned using various prediction tools. Five tools were mainly used: SignalP v4.1 for identification of Sec signal peptides [23], LipoP v1.0 for identification of lipoprotein signal peptides (SpII) [24], PredTAT for identification of TAT and Sec signal peptides [25] Phobius for prediction of signal peptide and TM domains [26], and TMHMM v2.0 for identification of TM domains [27]. The default prediction confidence threshold values of each prediction tool were used.

Conflicts between prediction tools were investigated through manual curation, taking into account the specificity of each tool, known homologues, existing literature, and the putative function of each protein based on common structural domains and functional motifs, as described in the InterPro [28], Pfam [29], and SMART [30] databases. The number of tools used to assign a subcellular protein location is included in the topology prediction score; thus, this score ranges from values of 0–5.

The content of SToPSdb is based on the published proteome of Uniprot and extended with 183 new ORFs that resulted from ongoing transcriptome-based genome annotation (Busche et al. in preparation). The content of SToPSdb and Uniprot is matched using Uniprot’s primary protein accession number and the gene name (format SLIV_[0–9]). Part of the annotation available in Uniprot, such as protein existence of structural domains are retrieved from Uniprot and retained in SToPSdb. Additional annotation, including the output of the protein prediction tools, annotation notes, references and experimental evidence are added to each protein and are available through SToPSdb. Annotation for protein function was added upon manual curation, based on the existence of known structural domains for each protein, as it is predicted in the InterPro [28], Pfam [29] and START [30] databases.

### Matching of the *S. lividans* and *S. coelicolor* proteomes

Comparative genome analyses was performed for genomes of *S. lividans* TK24 (NCBI accession number: NZ_CP009124) and *S. coelicolor* A3(2) (NCBI Accession Number: NC_003888) by EDGAR 2.0 [31] to identify orthologs in this genomes. For orthology estimation EDGAR 2.0 uses bidirectional bestBLAST [32] hits with a generic orthology threshold calculated from the similarity statistics of the compared genomes using the BLAST Score Ratio Value approach suggested by Lerat et al. [33].

### Database implementation

SToPSdb is a web-based database. The data is organized and accessed through the mySQL database management system. The web interface of SToPSdb was implemented and generated by PHPMaker, a PHP- and Javascript-based Content Management System [http://www.hkvstore.com/phpmaker/]. Visual interventions have been implemented with the jQuery library. IM topology graphs are plotted using the JpGraph Object-Oriented chart library (http://jpgraph.net/) and subcellular location distributions under the cell cartoon are drawn using the “Highcharts”. The architecture of SToPSdb is identical to the *E. coli* database, STEPdb, as previously described [22].

SToPSdb can be accessed through an online interface at http://stopsdb.eu with no user restrictions. The user can download all of the provided information via xls files in the separate “Downloads” section.

## Results

### Re-annotation of protein description

A significant limitation and challenge when studying less extensively characterized organisms is the large amount of proteins with generic names/descriptions (e.g. unknown protein, secreted protein etc.). In addition, public databases often use different generic terms without a consistent nomenclature, sometimes different terms are used within the same database. Before proceeding with any protein topology annotation in *S. lividans*, we re-annotated most of these protein names/descriptions to more specific annotations, based on sub-cellular topology and structural/functional signatures derived from their homologues and InterPro, Pfam or SMART databases (Additional file 1: Table S1). In many cases this information provides credible predictions for the protein’s function in the absence of any experimental evidence. A set of five rules was used to assign protein subcellular localization:

- Rule 1: If a protein contains a Sec/TAT or lipoprotein signal peptide and no TM, it is considered a secretory protein of the exportome.
- Rule 2: If a protein has at least one predicted TM region (or, optionally, a signal peptide sequence plus at least one TM), it is considered an integral membrane protein of the exportome.
- Rule 3: If there is no evidence for TM regions or signal peptides, the protein is defined as cytoplasmic.
- Rule 4: If a protein satisfies Rule 3 but experimental or homology data suggests that it can be secreted, then it is considered secreted. This is the case for some piggy-backing TAT substrates and for proteins identified in secreted vesicles (see below).
- Rule 5: if a protein is of high similarity with known proteins, then after curation it is assigned a more specific subcellular location (e.g. ribosomal, nucleoid, peripheral inner membrane, peptidoglycan binding).

In addition, manual curation enabled a more insightful annotation about their function and/or subcellular location for several proteins that have so far been assigned temporary generic names. We followed a defined format for protein nomenclature. Names/descriptions are divided into two parts separated by a hyphen, the first describing the subcellular localization group and the second the protein function. For cytoplasmic proteins, no topological suffixes are added. Integral membrane proteins are labeled as “Integral membrane protein –”, lipoproteins as “Secreted lipoprotein” and extracellular proteins, determined as described below, start with “Secreted protein”. For secreted proteins, we include in parenthesis the secretion system that is used for their translocation through the membrane [e.g. (Sec), (TAT) or (T7SS)]. In the second part of the name we include a description of the protein function (e.g. “Esterase”). For example, protein A0A076MHP6 (Uniprot accession) was re-annotated from “uncharacterized protein” to “DNA alkylation repair enzyme” because of its structural similarity with the proteins of this family, as it is described by InterPro, and protein A0A076M7D5 was re-annotated from “secreted protein” to “Secreted protein (Sec)—Solute-binding family 1 protein”.

In total, more than 4000 protein descriptions (~ 53% of the proteome) were re-annotated and are included in the database. For example, it has been reported that *S. coelicolor* A3(2) contains two genes encoding *Streptomyces* subtilisin inhibitor-like (SSI) proteins (genes SCO0762, SCO4010) [34]. For *S. lividans* TK24, the homologue of the second of these two genes was labeled in Uniprot as “uncharacterized protein”, however we assigned the re-annotated protein name based on structural similarity search in InterPro and matching with the homologous proteins of *S. coelicolor*. To help the user, all the re-annotated protein names/descriptions are presented in a downloadable table and online (see below) next to the current Uniprot name and a link to the relevant Uniprot WWW page. Additional representative examples or protein description re-annotation are included in Additional file 2: Table S2.

### Annotation of protein sub-cellular topology

**Fig. 1:**
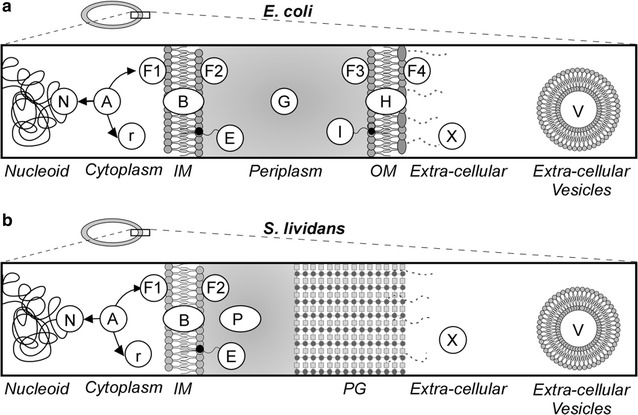
SToPSdb subcellular topology nomenclature. Single letter formalism in SToPSdb for the designation of the different subcellular compartments, corresponding to the equivalent GO term (see Additional file 3: Table S3) for both Gram-negative (a) and Gram-positive (b) bacteria. For Gram-positive bacteria, proteins are classified into 10 categories: N: nucleoid-associated, r: ribosomal, A: cytoplasmic, F1: peripherally associated with the membrane facing the cytoplasm, B: integral membrane proteins, E: secreted lipoproteins, F2: secreted peripherally associated proteins, P: peptidoglycan binding proteins; X: extracellular secreted proteins; V: extracellular vesicle proteins

Overall, SToPSdb aims to reduce the number of proteins with unknown topology, based on prediction tools, homologies and/or literature. A defined list of subcellular topologies for each protein is used, following the nomenclature previously presented in STEPdb [22], for the *E. coli* K-12 proteome annotation (Fig. 1a). The possible topological assignment for each protein corresponds to specific GO (gene ontology) terms. The GO terms are still considered as a main sub-cellular localization classifier for each protein but are less convenient for everyday use. Instead, for simplicity, we use a single letter formalism introduced in *Echo*LOCATION [35] and refined in STEPSdb [22] (Additional file 3: Table S3, Fig. 1b). For Gram-positive bacteria, the proteome is divided into 9 subcellular locations, a subset of the 13 subcellular locations found in Gram-negative bacteria (Fig. 1a). At the highest level, two basic groups can be identified, cytoplasmic and exported (exportome) proteins. Cytoplasmic proteins (one letter code: A) are further divided into Nucleoid (N), ribosomal (r), or Peripheral inner membrane proteins facing the cytoplasm (F1). Exported proteins use any of the appropriate secretion systems of the cell (see below) to become either integrated into the membrane (B; membranome) or to become completely translocated across it (secreted proteins; secretome). The secreted proteins are further separated into four classes. Secreted lipoproteins are peripherally anchored to the outer leaflet of the plasma membrane (after their secretion), via lipids covalently bonded to a cysteinyl residue at the N-terminus of the exported protein (E). Secreted peripheral membrane proteins interact with the outer leaflet of the plasma membrane through non-covalent interactions (F2) and have only been studied in some depth in *E. coli* [36, 37]. Peptidoglycan binding proteins (P) are secreted proteins that interact with the peptidoglycan layer, and extracellular (X) proteins are secreted beyond the peptidoglycan layer to the extracellular matrix. Dual or more topologies are not unusual, referred to as “moonlighting” [38]. Thus, a protein could be cytoplasmic, could bind to DNA and also to the membrane as a peripheral protein, e.g. the transcription regulator CsgD in *E. coli* [39] or typical cytoplasmic proteins, consistently found in spent growth media of *Streptomyces* such as TerE (D6EFX5) and SodF1 (D6ECW7) [40, 41]. Proteins with multiple topologies are separated by comma (e.g. A, r) in the relevant topology column of the protein lists in the database and proteins with undefined subcellular location are labeled with the letter “U”.

**Fig. 2:**
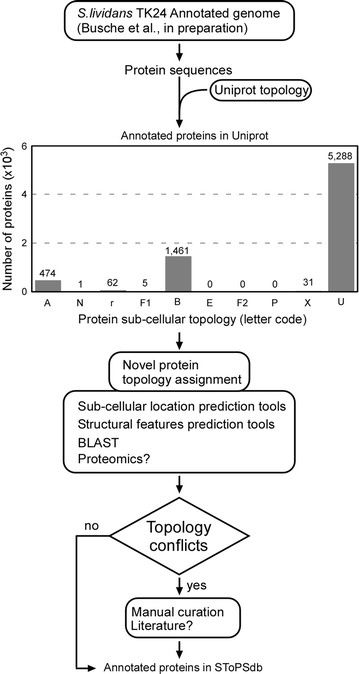
Overview of the topology annotation workflow. Protein sequences as derived after the re-annotation of *S. lividans* TK24 genome (Buche et al. in preparation) were used. The protein topology annotation in Uniprot was evaluated, revealing a large proportion of non-annotated proteins. De novo protein topology was assigned using prediction tools for topology or structural features followed by manual curation. Topology conflicts were manually curated. N: Nucleoid; r: Ribosome; A: Cytoplasmic; F1: Peripheral inner membrane protein facing the cytoplasm; B: Integral Membrane; F2 Peripheral inner membrane protein facing the periplasm; E: Inner Membrane Lipoprotein; P: Peptidoglycan binding; X: Extracellular; V: Extracellular vesicle; U: Uncharacterized

Initially, we evaluated the subcellular topology annotation available in Uniprot for the TK24 proteome. This was based on the genomic analysis and subsequent automated annotation (Fig. 2, Table 1; last update July 2017). Of the 7322 products of protein-encoding genes identified in the *S. lividans* proteome, 2153 proteins (29%) had been assigned GO (gene ontology) annotations for Cellular Compartment (CC) via automated proteome annotation programs. This number of proteins corresponds to 29% of the total proteome leaving the remaining 71% with undefined topologies.

**Table 1:** Summary of previously existing subcellular annotation and comparison with SToPSdb

	Proteome annotation	Unit of measure	Uniprot	SToPSdb
*S. lividans* TK24 (total proteome)		Number of proteins	7322	7505
Subcellular topology annotations	Existing	Number or proteins	2153	7494
		% of reference proteome	29%	>99%
	Contradicting topology annotations between Uniprot and SToPSdb	Number or proteins	12	12
	Missing/unresolved	Number or proteins	5169	11
		% of reference proteome	71%	< 1%

Approximately 30% of the *S. lividans* TK24 proteins contains proteome annotation for topology in Uniprot (2153 out of 7322). For 12 proteins Uniprot and SToPSdb were not in agreement. Protein topology for this strain was assigned de novo in SToPSdb leaving a small fraction of 11 proteins without topological annotation. Proteins annotated only in one of the two resources are labeled as “Unique (total)”

Next, we re-examined de novo the existing protein topology predictions in Uniprot for the 29% of the proteome using prediction tools and manual curation as described in “Methods” section. Topology conflicts between prediction tools were resolved by manual curation. A common example of topology prediction conflict is the mis-prediction of signal peptides as N-terminal TM domains. To resolve these problems we used SignalP version 4.0 that takes into account additional features including the length of the hydrophobic helix and the presence of a cleavage site [23]. Comparing the protein topology of the current version of Uniprot and SToPSdb, there are topology assignment differences for 12 proteins (< 1% of the annotated proteins in Uniprot) (Additional file 4: Table S4). 7 of these proteins were annotated as “integral membrane” and 2 of them as “extracellular” in Uniprot and converted to “cytoplasmic” in SToPSdb, due to lack of evidence of any integral membrane sequence, based on the prediction tools. 3 proteins were described as “integral membrane” in Uniprot and converted to extracellular or secreted lipoprotein in SToPSdb due to the prediction of Sec and Lipoprotein signal peptides. The reason for this discrepancy may be either the automatic protein annotation in Uniprot that may occasionally result in false positive hits, or the wrong prediction of an N-terminal TM domain instead of a signal peptide sequence. We resolved these conflicts by manually evaluating the output of each prediction algorithm used in this study, assigning the corresponding subcellular localization.

Subsequently, we proceeded with the annotation of the 71% of the proteome that had remained completely un-annotated in Uniprot. The comprehensive re-annotation of the *S. lividans* TK24 genome and transcriptome (Busche et al. in preparation) added 183 new protein-encoding ORFs, and this updated information has been included in SToPSdb. This brings up the total number of proteins in the *S. lividans* TK24 proteome to 7505 of which we provide topological annotation for the complete proteome (Table 1).

### Proteome-wide analysis of sub-cellular protein topology in *S.lividans*

**Fig. 3:**
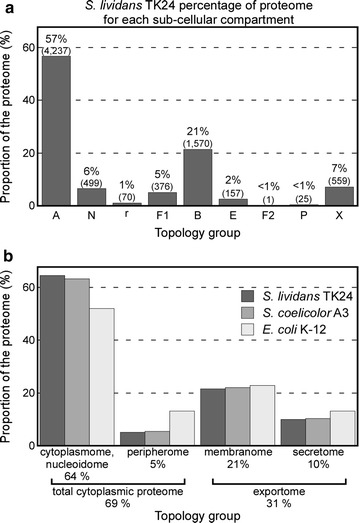
Distribution of proteins across the subcellular compartments. **a** Percentage and absolute numbers of proteins corresponding in each subcellular compartment in *S. lividans* TK24. **b** Summarized proportion of proteome corresponding in each topology group for the three model bacterial strains *S. lividans* TK24, *S. coelicolor* and *E. coli* K-12. Topology of the *S. coelicolor* proteome was extrapolated by the *S. lividans* homologues and additional bioinformatics analyses

Upon topological annotation of the complete proteome, 2312 proteins (29% of the total) are found to comprise the exportome and use the Sec, TAT or Type VII secretion pathways either for their insertion into the membrane or their complete translocation across it (Fig. 2). We separate the exportome into the integral membrane proteome or “membranome” (1571 proteins; 21% of the total proteome) and the secretome (proteins that are completely translocated through the membrane). The secretome (742 proteins; ~ 10% of the total proteome) includes secreted membrane-attached lipoproteins as well as extracellularly secreted proteins that use the Sec (581 proteins, 8% of total proteome), TAT (157 proteins, 2% of total proteome), or Type VII secretion pathways (3 secreted proteins known to date) (Fig. 3a).

### Proteome-wide analysis of protein sub-cellular topology in *S. coelicolor*

Having a completely annotated proteome for *S. lividans* we proceeded to deriving the proteome-wide topology for the closely homologous *S. coelicolor* strain A3(2), a main model of secondary metabolite production [10]. This was extrapolated based on the respective homologues in *S. lividans* following a proteome-wide BLAST analysis. The two proteomes are very similar and share 94% average nucleotide identity (ANI) for the matching sequence at the genome level and 93% of proteins at the protein sequence level, although *S. coelicolor* has a larger proteome. For the 1064 proteins in *S. coelicolor* that do not have *S. lividans* homologues, the localization prediction tools and manual curation were applied as above. Only 2571 out of 8038 proteins of *S. coelicolor* had available subcellular topology annotations in Uniprot. Following our re-annotation process 8029 proteins of *S. coelicolor* (> 99% of the total proteome) have now been assigned subcellular topologies.

Automated prediction of protein sub-cellular localisation for several Gram-positive bacteria is provided by an already established excellent tool—the LocateP database [21]. Upon comparison of protein localization curated in SToPSdb with that provided by LocateP, for *S. coelicolor*, 374 out of the 8038 proteins annotated in LocateP are in disagreement with SToPSdb and 254 are not directly comparable due to the different labeling of subcellular localization between the two databases (4.7 and 3.2% respectively) (see Additional file 5: Table S5). Most of the conflicts relate to integral membrane and exported proteins, for which the proportion of proteins with contradictory topology is 5.7 and 21.6%, accordingly. The discrepancy in subcellular localization assignment between the two databases can be attributed to the different type of prediction tools used by each of. To ensure accurate annotation we performed manual curation to each of them, resolving the conflicts between the prediction tools. LocateP has no predictions for the *S. lividans* proteome.

The generation of complete topological annotations allowed us to compare the two model *Streptomyces* proteomes with that of the well characterized model bacterium *E. coli* K-12. Similar proportions of each proteome comprise the cytoplasmic, integral membrane and fully secreted proteins between each of the two *Streptomyces* strains and *E. coli* K-12 (Fig. 3b).

In some cases, the same protein has a similar but distinct topology as is the case for FtsY that is an integral membrane protein in *Streptomyces* [42] but a peripheral protein in *E. coli*. In other cases, *Streptomyces* has two homologues of a protein like the peripheral membrane protein SepF [43].

### Protein secretion systems

Many of the components of the Sec secretion machinery of *S. lividans* TK24 have been annotated in Uniprot (Additional file 6: Table S6). Additional components were identified in SToPSdb, based on structural homology and sequence similarity with characterized proteins (e.g. SecG, YajC and a second homologue of the membrane protein insertase YidC). Single copies of SecA, SecY, SecE, SecG, that form the core essential Sec translocase [16], Trigger factor (TF), signal recognition ribonucleoparticle (SRP; Ffh protein component), and the SRP receptor (FtsY protein) were identified. SecD (A0A076M8T9) and SecF (D6EPR3), that are auxiliary components of the translocase, are present both as separate proteins, and in an additional form in which they are fused in a single polypeptide SecDF (D6EKP9), that was previously reported in *S. coelicolor* [44, 45]. Functionally, SecD and SecF, and the fused SecDF may function as membrane-integrated chaperones driving using the proton motive force (PMF) [46]. Two copies of the YidC insertase have been identified in *S. lividans* TK24, D6EMB4 (homologue of O54569 from *S. coelicolor*) and D6EWK6.

The TAT secretion system secretes folded proteins across the plasma membrane using their own characteristic signal peptides [17, 47]. 157 proteins (21% of the predicted TK24 secretome), are expected to be TAT-substrates. 32 of them had been previously experimentally described as TAT substrates in other *Streptomyces* strains [48–50]. Four proteins of TK24 that carry no predicted TAT signal peptide were additionally annotated as potential TAT-secreted substrates using a “piggy-back” mechanism [22]. Three of them were annotated after being homology matched to the YagG/R/S of *E.coli* K-12 (Identity > 37%, BlastP E-value < 5e−102). The fourth protein, tyrosinase MelC2, was experimentally shown to be exported in complex with its chaperone MelC1 using the TAT system [50]. Regarding the structural components of the TAT-secretion system, two functional copies of TatA and one each of TatB and TatC were identified, as seen in *S. coelicolor* [45, 47] (Additional file 6: Table S6).

The Type VII secretion system characterized in *Mycobacterium* and other Gram positive bacteria is also present in other *Actinobacteria*, including *Streptomyces* [51–53]. Nine T7SS proteins (six structural export machinery components and three secreted proteins) were annotated, based on their structural motifs and homologies. Seven of them were mentioned as uncharacterized proteins in Uniprot. Three of these proteins are ESAT-6-like (early secretory antigenic protein) secretory proteins common in *M. tuberculosis*, two are integral membrane serine proteases and four are similar to the structural components EccB, EccC, EccD, and EccE of the mycobacterial T7SS [51] (Additional file 6: Table S6).

Bacteria have evolved poorly understood mechanisms of “non-classical”, signal peptide-independent, secretion pathways [54–56]. One such mechanism is using extracellular vesicles [54]. *S. lividans* produces this type of vesicles that vary in their ultrastructure and macromolecular composition and arise at sites containing peptidoglycan layer defects. They may be related with acquisition of nutrients and virulence against other microorganisms [41]. 26 experimentally detected proteins (12 cytoplasmic, 3 lipoproteins, 11 extracellular secreted proteins) found in such vesicles are annotated in SToPSdb [40, 41].

### Mechanisms and properties that influence cell envelope protein topology

Additional proteins that influence protein subcellular topologies have also been annotated in SToPSdb. Several classes of sortases exist and depending on their class they recognize specific amino acid sequence motifs [57]. Four putative sortases were identified in *S. lividans* TK24, based on structural motifs, two of them are of class E [58, 59], commonly found in *Actinobacteria*.

Tail-anchored membrane proteins (TAMPs) [60] have a broad range of functions that are targeted to the membrane post-translationally through C-terminally located TMs sequence [16, 61, 62]. In many cases, no other N-terminal sequences apart from the C-terminal targeting sequences seem to be required and the C-termini alone can localize GFP to the membrane and the N-termini are non-conserved [60]. 73 such proteins have been predicted in the *S. coelicolor* proteome [60]. Of these, 16 have homologues in TK24.

Over-secretion of Sec substrates, could lead to detrimental accumulation of misfolded proteins in the membrane [63]. The CssRS two component system is activated upon secretion stress regulating HtrA-like proteases [64] for misfolded protein degradation [65]. Both the regulatory proteins (CssR, CssS) and the proteases (HtrA1-3, HtrB) have been included in SToPSdb.

Structural motifs can target proteins to the membrane. The conserved bacterial OsmY and nodulation (BON) domain, possibly targets proteins to membranes, through recognition of the phospholipid surface [66]. In *S. lividans*, 6 previously unannotated BON domain proteins, were annotated as Peripheral plasma membrane proteins facing the cytoplasm.

### Web interface and implementation

The web interface of SToPSdb can be accessed via the URL http://www.stopsdb.eu. On the top-right corner of the webpage there is a search box, allowing quick queries of specific proteins or advanced search options using gene name, Uniprot accession number, or protein name (see also [22]). On the left side of the website is the navigation panel containing three main sub-categories: “Strains”, “Proteomics” (see above) and “Downloads and Tools”; a link to the sister database of *E. coli* STEPdb is also provided. In the Strains panel, the two *Streptomyces* strains included in SToPSdb are listed; currently these include *S. lividans* TK24 and *S. coelicolor A3*(*2*). By clicking on the “*S. lividans* TK24” link, a table of proteins will appear on the right, containing the basic identifiers and the protein topology for each protein in this strain. Each protein is linked directly to Uniprot by clicking on the protein accession number. A “more info” button will open a tab with information about the manual curation process (e.g. topology score, references, notes), the prediction tools results, the identified protein family domains, and results of any applied biophysical property prediction tools. Different subsets of the secretory proteins can be selected from the Strains navigation menu. Each of these pages contains a table with the respective proteins and their characterization as described above. In addition, a comparative table between the *S. lividans* TK24 and *S. coelicolor* protein IDs and a table containing the *S. coelicolor* protein topology, as it is extrapolated from that of *S. lividans* based on homology, are also included.

The Downloads and Tools menu lists a series of links with supporting information and tools. The “SToPStoGO” page contains the protein topology nomenclature used in the SToPSdb and the corresponding GO terms. “IDMapping” is a tool that reads a list of protein accession numbers returning the corresponding topology. The full content of the database can be found in the “Downloads” page and is available to any user with no restriction. Each protein contains a Uniprot accession number that links SToPSdb with Uniprot, allowing the direct comparison of the SToPSdb entries with the reference proteome database. In addition, the SToPSdb subcellular localization labeling is translated to the corresponding GO terms for cellular compartment, retaining the commonly recognized rules for protein annotation [67, 68]. Information about terminology, tools and annotation rules used in SToPSdb server are included in the “About” page.

### Proteomics data

The exportome of *Streptomyces* is of core interest and has attracted a significant effort in SToPSdb. Given the importance of experimentally detected/validated proteins in the annotation process, SToPSdb incorporates publicly available experimental data for proteins detected in the exportome (“Proteomics” panel divided in “exportome” and “secretome”). This information can also be downloaded from the “Downloads” page, and the corresponding reference for each study is listed for more detailed datasets. Experimentally detected proteins are listed in one table, and the experimentally detected proteins are marked in the column corresponding to the relevant study. Because the culture medium composition can considerably change the profile of the secreted proteome and has been a focus of many studies [19, 69, 70], the media used in each study are indicated. Until now experimentally validated TAT substrates (31 proteins) [48, 49], proteins detected in extracellular vesicles (26 proteins) [40, 41] or in the exportome of *S. lividans* TK24 growing in MM–CAS medium (296 proteins) [71], have been presented. An additional comparative proteomics study between *S. lividans* and *S. coelicolor* growing under low O_2_ conditions, detected 1832 proteins from which 1486 are cytoplasmic and 346 exported proteins [72].

## Discussion

We developed SToPSdb to provide information on the protein topology annotation of *Streptomyces*, using as a model organism *S. lividans* TK24 [7]. SToPSdb is an extension of STEPdb (http://www.stepdb.eu), a database for the comprehensive topological annotation of the Gram-negative model *E. coli* K-12 [22]. Development of these databases stemmed from the realization that proteomes of critical model microorganisms of biological, biomedical or industrial interest, are still poorly annotated. Moreover, despite the substantial recent progress in automatic annotation, it is obvious that such tools are not yet capable of providing comprehensive and accurate annotations for whole proteomes. This leaves a pressing need for critical manual curation on top of a judicious use of multiple bioinformatics tools. Comprehensive annotation is necessary for all omic-level studies.

An example of the beneficial outcome of such curation is the annotation of proteins involved in the secretory pathway. The YidC insertase, with distinct functions and low sequence identity, has been reported only in some Gram-positive bacteria [73, 74]. The precise function of the second homologue and whether it specializes in the membrane integration of particular substrates remains to be determined. Another group of proteins of potential industrial interest for site-specific protein tagging are the Gram-positive sortases that covalently link secreted proteins with peptidoglycan and control cell shape [75]. Finally, TAMPs in TK24, are tail-anchored membrane proteins that have no predicted Sec or TAT signal peptide and unknown function that awaits future study.

Currently, automated annotation for *Streptomyces* is provided through the is LocateP database [21]. Comparison of LocateP and SToPSdb for *S. coelicolor* showed conflicting topological annotation mainly for secreted proteins. The disagreement between these two databases can be attributed to the use of different prediction tools or versions of the same tool. In addition, automated prediction tools will allow a proportion of misclassified proteins, in order to avoid overfitting and generalize well. On the other hand, manual curation can correct more misclassified proteins, but is limited to a small number of organisms that can be annotated.

Emphasis was also given in SToPSdb on the connection of *S. lividans* TK24 annotation with that of other model strains including *S. coelicolor* and *E. coli* K-12, based on their homologous proteins. One interesting observation here is that despite the significant phylogenetic differences between *Streptomyces* and *E. coli*, their differences in proteome size, transcription regulation diversity and developmental capacities, the percentage of each proteome devoted to cytoplasmic (~ 69%) or exportome (~ 31%) proteins remains rather similar (Fig. 3). The higher percentage of *E. coli* proteins identified in the peripherome may reflect the dearth of available experimental data in *Streptomyces* for this biochemically unusual class, rather than a true numerical difference.

We expect SToPSdb to become a reference resource for researchers working with *S. lividans,* in particular, and streptomycetes in general. We plan to link SToPSdb with integrated omic databases and reconstructed metabolic and protein network models that are currently being developed. This will enhance a comprehensive analysis of the functional processes of *S. lividans* and can assist in further genetic engineering and biotechnology efforts that can be rationally applied. Towards this direction, we have integrated in this first iteration of the database experimental data derived from proteomics analysis of the exportome. These were derived from multiple proteomics studies that focused on various modes of protein export [41, 48, 49, 71].

## Conclusions

Improvement of the annotation of *Streptomyces* is an important step towards understanding the complexity of this genus of bacteria and its rational exploitation. SToPSdb provides a proteome annotation database focusing on protein topology. Although we use *S. lividans* TK24, as the model strain the proteome annotation is linked also to *S. coelicolor* and *E. coli* K-12, based on homologous proteins. This effort lays the foundation for future developments that will include the connection of SToPSdb with other resources containing experimental information at various physiological conditions, providing a reference resource for this organism. SToPSdb can be easily accessed through a web interface at http://stopsdb.eu.

## Additional files

**Additional file 1: Table S1.** Re-annotated protein names and Identifiers for the proteins included in SToPSdb.

**Additional file 2: Table S2.** Additional examples of protein description re-annotation in SToPSdb.

**Additional file 3: Table S3.** Protein topology nomenclature.

**Additional file 4: Table S4.** Conflicts in protein topology between Uniprot and SToPSdb.

**Additional file 5: Table S5.** Comparison of SToPSdb and LocateP topological annotation.

**Additional file 6: Table S6.** Secretion system components in *S. lividans* TK24.

## Abbreviations

ANI: average nucleotide identity
CC: cellular compartment
ESAT: early secretory antigenic protein
GO: gene ontology
MM–CAS: minimal medium–casamino acids
PMF: proton motive force
SP: signal peptide
SRP: signal recognition particle
SSI: *Streptomyces* subtilisin inhibitor
SToPS: Subcellular Topology of Polypeptides in Streptomyces
TAT: twin-arginine translocation
TAMPS: tail-anchored membrane proteins
TM: transmembrane
T7SS: type VII secretion system
